# Implications of sex offender classification on reporting demographic characteristics, health, and criminal careers: results from an Australian jurisdiction

**DOI:** 10.1186/s12874-020-00960-w

**Published:** 2020-04-28

**Authors:** Mathew Gullotta, David Greenberg, Armita Adily, Jesse Cale, Tony G. Butler

**Affiliations:** 1 Kirby Institute, University of New South Wales, Sydney, Australia; 2 Statewide Community Court Liaison Services, Sydney, Australia; 3 School of Psychiatry, University of New South, Sydney, Australia; 4 School of Social Sciences, University of New South, Sydney, Australia

**Keywords:** Adult sex offender, Child sex offender, Classification, Data linkage, Health, Polymorphous, Specialisation

## Abstract

**Background:**

Cross-sectional and retrospective offence data are often used to classify sex offenders in epidemiological and survey research, but little empirical evidence exists regarding the practical implications of this for applied research. This study describes the classification of sex offenders from a cohort of prisoners recruited as part of an Australian inmate health survey and the implications for reporting results.

**Methods:**

Data-linkage was used to join the New South Wales (NSW) Inmate Health Surveys to the states re-offending database to identify men with histories of sexual offending. Sex offenders were classified into men who sexually offended against children only (ChildSOs), against adults only (AdultSOs), and men who sexually offended against both children and adults (Age-PolySOs).

**Results:**

Using historical offending data rather than the current offence information only, an additional 35.4% of men with histories of sexual offences were identified. Differences were found between the three sex offender subgroups in terms of demographic characteristics, health, and criminal careers. Age-PolySOs reported higher educational attainment, were less likely to report being self-employed, single marital status, and having children. Half the ChildSOs self-reported a mental health issue and half of the ChildSOs and Age-PolySOs reported four or more chronic health conditions. Age-PolySOs were older than the other sex offender groups when committing their first non-sexual, non-violent crime (*M* = 43.2 years, *SD* = 13.8); violent crime (*M =* 39.5 years, *SD* = 11.1); and sexual crime (*M =* 47.8 years, *SD* = 11.2). Age-PolySOs also committed more sexual offences (*M =* 5.91, *SD* = 11.2) compared to those who only offended against one victim age group.

**Conclusion:**

These findings suggested that historical offending records should be used to more accurately identify sex offender subgroups and that differences in demographic, health, and criminal careers exist for the different sex offender subgroups.

## Background

Sex offenders have been studied extensively by researchers from disciplines including criminology, psychology, and psychiatry. The focus of that research includes describing demographic information [1], criminal careers [2] including recidivism, psychological and psychiatric aspects [3], risk assessment, and the management and treatment of sex offenders [4].

Criminological and health research on specific offender groups such as sex offenders requires classifying individuals using either official records - from the police, corrective services departments, and/or the courts - or self-reported offending [5]. The index offence (also referred to as the most serious or cardinal offence) for the current episode of incarceration is commonly used as the basis for classifying offenders into different groups based on offence type. This can be problematic as it focuses on an offence at a single point in time and does consider other crimes which may have been committed in the past, potentially overlooking those with serious historical crimes such as sexual offences. Furthermore, the most serious offence reflects a subjective political view of sentencing and there does not appear to be a standardized or universally accepted hierarchy of offence seriousness.

Men who commit sexual offences are often classified into one homogeneous group: ‘sex offenders’. This lumping together of those who have commited a sexual offence into one amorphous group may occur due to small sample sizes, limited access to historical offending data, or for convenience. Notwithstanding the theoretical implications, this practice can result in important differences between different sex offender subgroups being overlooked and potentially incorrect conclusions being drawn, depending on the focus of the research [6, 7].

Psychological (and more recently criminological) research has produced three broad groupings of studies that have classified male sex offenders into subgroups [5, 8]. The most common classification differentiates between men who commit sexual offences against children (ChildSOs), men who commit sexual offences against adults (AdultSOs), and those who switch between age groups (age-polymorphous – (Age-PolySOs)). ChildSOs and AdultSOs have been studied extensively and differences between these two groups are well documented in terms of their  demographic characteristics and the nature and extent of criminal behaviour (e.g., age of offending onset, frequency, versatility and specialisation of offending) [9–11]. ChildSOs often achieve higher academically and professionally, have different social and intimate relationships, and are less likely to abuse alcohol and substances than AdultSOs [9, 12]. ChildSOs are also likely to have a later onset for sexual offending – in part to do with more successfully evading detection, and also to unique offending opportunity structures that present later in life - tend to be less versatile in their offending (i.e., commit only few different types of crime); sexually offend for longer periods (i.e., from age of onset to age of desistance); and, have more victims than AdultSOs [2, 13].

Much less is known about men who commit sexual offences against multiple victim age groups (Age-PolySOs). Empirical studies have shown that approximately 25% [14] to 89% [15] of sex offender samples contain men who sexually offend against both children and adults, depending on the methodology employed (i.e., official records vs. self-report). While there are other forms of sexual polymorphism (e.g., victim-gender, victim-offender-relationship, and the nature of acts committed), age-polymorphism appears to be the most common [16]. Age-PolySOs have been found to be more likely to reoffend sexually (43%) compared to ChildSOs (3%) and AdultSOs (19%) after a 15-year follow-up [17]. However, a recent study found that this relationship appears to be mediated by the number of victims [18]. Nevertheless, Age-PolySOs may be unique to other sex offender subgroups in terms of their demographic characteristics, physical and mental health histories, and criminal careers. Little research has focused on differences in these characteristics for this group.

While identifying and describing the demographic and clinical characteristics of different subgroups of sex offenders is important, historically clinical research along these lines has not been theoretically informed. Furthermore, from a criminal careers perspective, the classification of men who commit sexual offences should consider: the onset of offending, a qualitative and quantative course of development (frequency of offending, escalation, specialization or versatility), and desistance (cessation of offending) [2]. These criminal career dimensions highlight the longitudinal pattern of offending over time. This is a useful development in the field because it allows for the identification of factors and outcomes associated with different courses of offending over the life-course. This represents the developmental and life-course criminological theoretical approach and can be contrasted with other clinical and theoretical models which aim to describe the propensity to commit sexual crime [19, 20]. Importantly, it also provides a framework for examining different types of sexual offending and whether such distinctions are relevant clinically and/or to policy.

Overall, there is no consensus as to how sex offenders should be classified. Little research exists on how different sex offender classifications can impact on the results of epidemiological and social research. Identifying the physical health, mental health, and health risk behaviours, such as substance abuse, as risk factors for specific sexual offender groups may have significant implications for: clinical practice in terms of developing specialised assessments and tailored treatment programs; criminal justice and health policy as resources could be directed to specific groups in need; and research.

This study describes the data-linkage process and classification of sex offenders in a cohort of prisoners recruited from three waves of an Australian inmate health survey. The health survey data was linked to the states re-offending database to identify all men in the cohort with historical sexual offences. This study also describes differences in the demographic  characteristics, health, and criminal careers of three sex offender subgroups (ChildSOs, AdultSOs, and Age-PolySOs).

## Method

### Data sources

This study used a retrospective cohort design that involved data-linkage of three waves of the New South Wales (NSW) Inmate Health Surveys conducted in 1996 [21], 2001 [22] and 2009 [23] to the NSW Bureau of Crime Statistics and Research’s Re-offending Database (RoD) [24]. The Inmate Health Surveys are described in detail elsewhere [21–23]. Briefly, the surveys recruited random samples of men and women prisoners stratified by age, sex, and Indigenous status from all NSW correctional centres. Participants were included if they were over the age of 18 years, spoke sufficient English to participate in the interview, and were able to provide informed consent. Face-to-face interviews were conducted in 1996 and 2001, and a combination of face-to-face and telephone interviews were conducted in 2009. The surveys were wide ranging: covering mental and physical health as well as health risk behaviours and included serological and urine screening for infectious diseases and sexually transmissible infections. The most serious offence for the custodial episode when the survey was conducted was recorded.

The Re-offending Database holds records of all finalised court matters and full-time prison episodes for offenders in NSW from 1994 to present [24]. For this study, data from all finalised court matters were accessed. The offence histories were coded according to the Australian and New Zealand Standard Offence Classification [25].

Approval for use of the survey data was provided by the Justice Health and Forensic Mental Health Network (JH&FMHN) and for offending data by the NSW Bureau of Crime Statistics and Research. Ethics approval for the data-linkage was provided by the JH&FMHN Human Research Ethics Committee (G70/14).

### Data linkage procedure

Data from the 2327 men who participated in the surveys were combined into one dataset.^1^ There were 213 men that participated in more than one of the surveys. When this occurred, the data from the earliest survey was used to capture the characteristics of the sample at the first time point of testing and to ensure equality in cross-sectional measurement independent of changes over time (in terms of demographics, health, and criminal careers). This resulted in a cohort of 2114 men. Deterministic linkage using a unique prisoner identifier or common identifiers (name, sex, and date of birth) matched the survey data to offending data extracted from the Re-offending Database. Offending data for 1853 (87.7%) of the cohort were available from January 1994 to October 2014.^2^

### Classification procedure

The cohort was classified into groups based on the type of crimes identified by both the survey and offending data from the date of the first available offence until the date of participation in the survey. Participants were classified as sex offenders if any *Sexual assault and related offence* [25] was recorded in their offence histories before or at the time of the health survey. Only contact sexual offences were included (aggravated sexual assault and non-aggravated sexual assault). Sex offenders were classified into subgroups based on victim age using the age of consent in NSW resulting in: child sex offenders (ChildSOs) that had sexual offences against only victims under the age of 16 years old; adult sex offenders (AdultSOs) that had only victims 16 years and above; and age-polymorphous sex offenders (Age-PolySOs) that had both children and adult victims. Violent (non-sex) offenders included those with records of *Homicide and related offences*, *Acts intended to cause injury*, *Abduction, harassment, and other offences against the person*, and *Robbery, extortion and related offences* [25] before or at the time of the survey. Other (non-sex, non-violent) offenders were those with crimes that did not fall into either sexual or violent crime categories.

### Measures

#### Demographic characteristics and summary health variables

Using the survey data, we examined demographic and descriptive characteristics of the sample including: age; Indigenous status (0 = *no*, 1 = *yes Indigenous*); less than high school education (0 = *no*, 1 = *yes*); usual occupation as self-employed (0 = *no*, 1 = *yes*), employed in the 6 months prior to imprisonment (0 = *no*, 1 = *yes*); marital status as single (0 = *no*, 1 = *yes*); children (0 = *no*, 1 = *yes*); first time in prison (0 = *no*, 1 = *yes*); and number of previous imprisonments. Juvenile histories were examined and included: parental imprisonment (0 = *no*, 1 = *yes*); ever placed in care (0 = *no*, 1 = *yes*); juvenile detention (0 = *no*, 1 = *yes*); age at first juvenile detection; and number of juvenile detentions. Summary health variables were also examined and included self-reported: mental health issue(s) (0 = *no*, 1 = *yes*); sexually transmissible infection(s) (0 = *no*, 1 = *yes*); number of chronic health conditions (0 = *none,* 1 = *one,* 2 = *two,* 3 = *three,* 4 = *four or more*); and overall physical and mental wellbeing in the past 4 weeks using the Short-Form Health Survey (SF-12) which is a reliable and valid 12 item measure of overall mental and physical well-being [26].

#### Criminal career parameters

Using the survey and offending data, we examined the age at first crime, frequency, and variety/specialisation for any crime, non-sexual, non-violent crimes, violent (non-sexual) crimes, and sexual crimes. Variety of offending was calculated on a scale from 1 to 3 by examining the presence of the following crime types: sexual crimes; violent (non-sexual) crimes; and non-sexual, non-violent crimes. Higher scores indicate higher offence variety. That is, men charged only for a sexual crime were scored with a 1 (indicating no variety in the offending history), while men with records of all three crime types received a score of 3 (indicating the highest level of variety). Specialisation for each crime category (i.e., sexual; violent; and non-sexual, non-violent) was calculated as a ratio of the number of charges in the respective crime category to the total number of charges in their offending history, and then multiplied by 100 to reflect a percentage.

### Statistical analysis

An exploratory approach was adopted to investigate differences between types of sex- and non-sex offenders in terms of demographic and criminal career parameters. Pearson’s Chi-square tests were used for categorical variables. One-way Analysis of Variance and *t-*tests were used for continuous variables. The Scheffe test was used for post-hoc comparisons because it is a conservative procedure that allows the examination of differences among groups, despite unequal group sizes. When the equality of variance assumption was not met, Tamhane’s T2 tests were used. Kaplan-Meier estimates were used to examine re-offending after the custodial episode in which the survey was completed to the date of the next recorded offence for each of the groups. There was no fixed follow-up time. Cox-regression survival analysis was used to estimate the association between offender type and re-offending. An a priori alpha level of α = .05 for each statistical analysis was used. Data were analysed by using IBM® Statistical Package for the Social Sciences, version 24.

## Results

### Offender classification

**Fig. 1:**
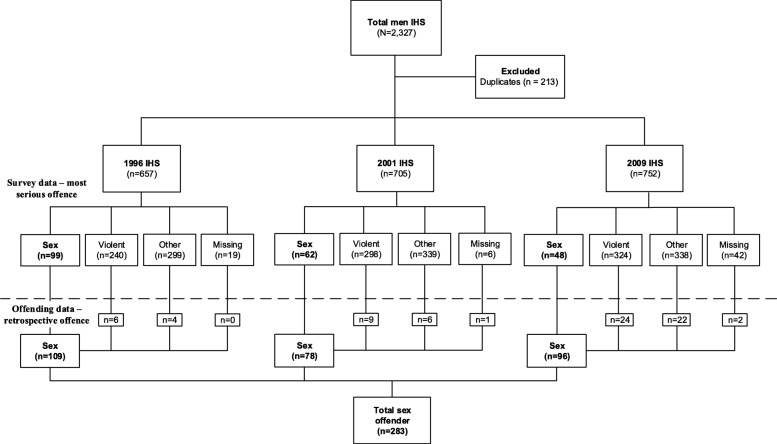
Data-linkage schema for men with sexual offences. *Note:* The surveys were separated by date to show the number of participants with sexual offences at the time of each survey iteration and before the survey as identified by the linkage to offending data

Figure 1 presents the data-linkage schema separated for the three health surveys to show the number of men with sexual offences identified at the time of each survey (above the broken line) and using offending histories to identify sexual offences committed before the survey (below the broken line). At the time of the surveys, 209 participants were identified as having a most serious offence that was a sexual offence. After performing the linkage, a further 74 participants were identified as having a historical sexual offence, giving a total of 283 sex offenders (35.4% increase). Similarly, an increase in the number of Violent offenders was found using this look-back procedure. At the time of survey, 862 participants were identified as Violent (non-sex) offenders but after the linkage, a total of 1269 Violent offenders were identified. For Other (non-sexual, non-violent) offenders, there were 976 that had another offence at the time of the survey but after the linkage, this decreased to 552 Other offenders. This suggests that offenders with histories of sexual and violent crimes were incarcerated for non-sexual, non-violent crimes at the time of the survey.

Sex offenders were classified into subgroups based on victim age which resulted in: 77 participants that had sexual offences against victims only under the age of 16 years (ChildSOs), 160 that had victims only above the age of 16 years (AdultSOs), and 43 that had sexual offences with both children and adults (Age-PolySOs). Three participants were removed from analyses as they only had child pornography offences on their records and no violent (non-sexual) or non-sexual, non-violent offences.

The juvenile sexual offending histories of the sample were examined. Twelve participants committed at least one sexual offence prior to the age of 18 years: three were identified to be ChildSOs and nine AdultSOs. We sought to remove the men who only committed sexual offences during their juvenile years as they are thought to represent a unique group compared to those who commenced offending or continued to sexually offend throughout adulthood [8]. Two of the ChildSOs committed another sexual offence after they were 18 years old against a child with whom they had more than a three to five-year age difference and were kept in the analyses, while the other only committed the one sexual offence in their juvenile years and was removed. Of the AdultSOs, two committed their first sexual offence (when 15 and 17 years old, respectively) against a person 16 years or over with further sexual offences against adults after they were 18 years old, two sexually offended at least twice before the age of 18 years against an age-related peer (victim over the age of 10 but under 16), and five only committed one sexual offence before the age of 18 years. The two who reoffended during adulthood were kept in the analyses while the other seven AdultSOs were removed.

### Comparing survey to historical sex offenders

Sex offenders identified at the time of the survey based on the index or most serious offence (‘survey sex offenders’) were compared to those identified by their historical offending records (‘historical sex offender’; Table 1). Relative to the historical sex offenders identified by their records, survey sex offenders were older  at the time of the survey, less likely to have been previously imprisoned or report juvenile histories of out of home care and juvenile detention, and had different health needs. For example, the survey sex offenders were more likely to report four or more chronic health conditions (39.2%) relative to their historical sex offender peers (20.5%), a finding which may reflect their age. Most of these differences appear to be attributable to differences between the ChildSOs identified at the time of the survey and the ChildSOs identified by their historical offending records. Few differences were found between the survey AdultSOs and historical AdultSOs.

**Table 1:** Demographic characteristics and summary health variables comparing survey and historical sex offenders

	Survey sex offender (any) (*n* = 209)	Historical sex offender (any) (*n* = 74)	*t* (df); X2(df)	Survey ChildSO (*n* = 55)	Historical ChildSO (*n* = 22)	*t* (df); X2(df)	Survey AdultSO (*n* = 110)	Historical AdultSO (*n* = 50)	*t (df); X*^2^(*df*)
Sample members (%)	73.9%	26.1%	–	71.4%	28.6%	–	68.8%	31.2%	–
Demographic characteristics									
Age at survey *M* (*SD*)	43.19 (13.95)	33.73 (12.01)	*t*(278)= 5.147***	47.99 (12.12)	34.84 (12.73)	*t*(73)= 4.159***	33.48 (11.95)	37.36 (13.29)	*t*(158)= 1.776
Aboriginal or Torres Strait Islander	31.1%	52.1%	*X*^2^ = 10.246**	23.6%	52.4%	*X*^2^ = 5.812*	36.4%	52.9%	*X*^2^ = 3.941*
Less than high school education	58.6%	60.96%	*X*^2^ = .085	67.3%	66.7%	*X*^2^ = .003	62.1%	57.1%	*X*^2^ = .346
Usually self-employed	38.0%	50.8%	*X*^2^ = 2.930	35.7%	68.8%	*X*^2^ = 5.113*	44.0%	42.9%	*X*^2^ = .016
Employed prior to prison	53.2%	44.4%	*X*^2^ = 1.639	53.7%	23.8%	*X*^2^ = 5.452*	51.9%	54.0%	*X*^2^ = .058
Marital status (single)	49.5%	58.3%	*X*^2^ = 1.662	34.5%	52.4%	*X*^2^ = 2.023	63.0%	60.0%	*X*^2^ = .127
Any children	60.5%	58.8%	*X*^2^ = .055	71.1%	76.2%	*X*^2^ = .186	67.5%	51.1%	*X*^2^ = 3.412
1st time in prison	56.3%	32.4%	*X*^2^ = 11.944**	78.8%	47.6%	*X*^2^ = 6.928**	42.3%	26.5%	*X*^2^ = 3.547
# of prior imprisonments *M* (*SD*)	2.64 (3.01)	3.38 (12.01)	*t*(268)= −1.830	1.44 (1.06)	3.00 (2.79)	*t*(71)= −3.480**	3.57 (3.49)	3.45 (2.69)	*t*(151)= −.212
Juvenile history									
Parent sent to prison^1^	9.1%	21.0%	*X*^2^ = 5.075*	14.3%	27.8%	*X*^2^ = 1.267	9.1%	18.2%	*X*^2^ = 1.771
Ever placed in care	16.7%	30.6%	*X*^2^ = 6.248*	20.8%	42.9%	*X*^2^ = 3.725	17.8%	26.0%	*X*^2^ = 1.427
Juvenile detention	21.0%	47.9%	*X*^2^ = 18.772***	9.6%	38.1%	*X*^2^ = 8.289**	29.5%	53.1%	*X*^2^ = 7.939**
Age at 1st juvenile detention *M* (*SD)*	14.39 (2.61)	13.56 (2.36)	*t*(58)= 1.294	15.60 (1.67)	12.13 (2.90)	*t*(11)= 2.415*	14.04 (1.99)	14.59 (2.67)	*t*(39)= .752
# times in juvenile detention *M* (*SD)*	3.26 (3.25)	4.90 (6.24)	*t*(56)= −1.231	2.00 (1.73)	7.38 (9.53)	*t*(11)= −1.229	3.13 (2.500)	4.04 (4.60)	*t*(158)= −.726
Summary self-reported health									
Any intravenous drug use	40.4%	63.9%	*X*^2^ = 5.651*	40.0%	81.8%	*X*^2^ = 4.547*	45.9%	58.3%	*X*^2^ = 1.065
Mental health issue(s)	35.9%	49.3%	*X*^2^ = 4.088*	40.0%	81.0%	*X*^2^ = 10.202**	30.9%	37.3%	*X*^2^ = .635
Sexually transmissible infection(s)	44.2%	37.1%	*X*^2^ = .887	42.1%	52.6%	*X*^2^ = .566	54.1%	30.2%	*X*^2^ = 5.824*
Number of chronic conditions:									
None	22.5%	30.1%	*X*^2^ = 10.310*	16.4%	33.3%	*X*^2^ = 4.513	30.0%	27.5%	*X*^2^ = 7.688
One	17.7%	23.3%		18.2%	9.5%		17.3%	29.4%	
Two	13.4%	12.3%		1.8%	4.8%		19.1%	15.7%	
Three	7.2%	13.7%		9.1%	14.3%		6.4%	13.7%	
Four or more	39.2%	20.5%		54.5%	38.1%		27.3%	13.7%	
SF-12 rating: Fair/poor	28.9%	33.3#	*X*^2^ = .792	35.2%	33.3%	*X*^2^ = .852	22.1%	34.0%	*X*^2^ = 2.520

*Note:* The information in this table is based the self-reported survey data. The sample size in this table reflect the number of participants who were able to be coded on each item. Some responses could not be coded because of the amount and quality of the information. The sample sizes ranged from Survey sex offenders 121–209; Historical sex offenders: 62–74; Survey ChildSOs: 28–55, Historical ChildSOs: 18–22, Survey AdultSOs: 55–110, Historical AdultSOs: 44–50

^1^ Data from 2001 and 2009 surveys

^*^p < .05, ^**^p < .01, ^***^p < .001

### Demographic characteristics

The offender groups differed on a number of demographic and descriptive characteristics (Table 2). There was a significant overall difference in age at time of survey. Post-hoc comparisons showed that Age-PolySOs were the oldest of all groups (*p*s ≤ .015), ChildSOs were older than the remaining groups (*p*s ≤ .001), and while AdultSOs and Other offenders were similar in age (*p* = 1.000), they were both older than violent offenders (*p*s < .001). Aboriginal and Torres Strait Islanders were over-represented in the AdultSOs group (39.6%). ChildSOs (69.9%) and Age-PolySOs (59.5%) had the highest proportion of men for whom this was their first prison sentence. There was a significant overall difference in the average number of prior imprisonments. With the exception of Age-PolySOs (*p* = 1.000), ChildSOs had the least number of prior imprisonments compared to the other groups (*p*s < .001). In terms of juvenile histories, Age-PolySOs were the least likely of the groups to report out of home care as a juvenile and being sent to juvenile detention. No differences we found between any of the groups in terms of age at first juvenile detention episode or the number of times they were placed in juvenile detention.

### Summary health variables

ChildSOs (51.3%) were the most likely group to self-report a mental health issue, while AdultSOs (33.8%) and Other offenders (26.8%) were the least likely (Table 2). Age-PolySOs were least likely to report sexually transmissible infections. Half of the ChildSOs and Age-PolySOs reported four or more health conditions, which likely reflects their older age than the other offender groups.

ChildSOs and Age-PolySOs tended to rate their health as “poor” on the first item of the SF-12 compared to the other groups, although this difference was not significant (Table 2). Scores of overall physical wellbeing on the SF-12 differed between the groups, *F* (4,2003) = 4.376, *p* = .002. However, post-hoc comparisons indicated that this difference was attributable to only a two point difference between ChildSOs (*M =* 51.07, *SD* = 9.70) and Other offenders (*M =* 53.97, *SD* = 7.21)(*p* = .050) which was trending toward being statistically significant and is not considered as meaningful. Overall, mental wellbeing was significantly different between the groups, *F* (4,2003) = 3.736, *p* = .005. This difference was attributable to a less than two point difference between Violent (*M =* 43.41, *SD* = 8.88) and Other offenders (*M =* 41.87, *SD* = 7.96)(*p* = .019).

**Table 2:** Demographic characteristics and summary health variables

	Total Sample (*n* = 2106)	ChildSO (*n* = 76)	AdultSO (*n* = 153)	Age-PolySO (*n* = 43)	Violent (*n* = 1269)	Other (*n* = 552)	*F (df); X*^2^(*df*)
Sample members (%)	–	3.6%	7.3%	2.0%	60.2%	26.2%	–
Demographic characteristics							
Age at survey *M* (*SD*)	33.74 (12.25)	44.30 (13.58)	36.73 (12.93)	51.78 (10.93)	30.88 (10.72)	36.63 (12.44)	*F*(4, 2091) = 73.403***
Aboriginal or Torres Strait Islander	30.3%	31.6%	39.6%	25.6%	35.5%	15.9%	*X*^2^ = 77.177***
Less than high school education	58.9%	67.1%	60.7%	42.9%	61.9%	51.6%	*X*^2^ = 22.646***
Usually self-employed	45.9%	44.8%	43.3%	27.8%	52.7%	32.1%	*X*^2^ = 51.188***
Employed prior to prison	50.3%	45.3%	52.4%	52.4%	48.9%	53.4%	*X*^2^ = 4.063
Marital status (single)	61.0%	39.5%	61.6%	35.7%	65.4%	55.5%	*X*^2^ = 43.101***
Any children	57.8%	72.7%	62.6%	32.4%	54.4%	65.0%	*X*^2^ = 31.254***
1st time in prison	36.7%	69.9%	37.7%	59.5%	32.8%	38.9%	*X*^2^ = 53.022***
# of prior imprisonments *M* (*SD*)	3.18 (3.04)	1.89 (1.86)	3.54 (3.31)	2.17 (2.83)	3.30 (3.14)	3.05 (2.82)	*F*(4, 2014) = 5.722***
Juvenile history							
Parent sent to prison^1^	17.1%	19.6%	10.9%	2.8%	20.4%	9.7%	*X*^2^ = 26.981***
Ever placed in care	21.5%	27.0%	21.3%	7.1%	25.5%	12.5%	*X*^2^ = 44.687***
Juvenile detention	37.5%	17.8%	34.7%	11.9%	44.3%	27.4%	*X*^2^ = 71.019***
Age at 1st juvenile detention *M* (*SD)*	14.08 (2.08)	13.46 (2.99)	14.34 (2.43)	12.80 (3.03)	14.05 (2.05)	14.35 (1.94)	*F*(4, 602) = 1.267
# times in juvenile detention *M* (*SD)*	4.50 (6.36)	5.31 (7.84)	3.61 (3.94)	5.40 (5.77)	4.82 (6.82)	2.94 (3.47)	*F*(4, 572) = 1.726
Summary self-reported health							
Any intravenous drug use	68.0%	57.7%	48.8%	14.3%	73.3%	63.1%	*X*^2^ = 48.870***
Mental health issue(s)	38.6%	51.3%	33.8%	39.5%	43.5%	26.8%	*X*^2^ = 51.942***
Sexually transmissible infection(s)	41.2%	45.6%	46.4%	32.4%	38.5%	47.1%	*X*^2^ = 11.349*
Number of chronic conditions:							
None	35.4%	21.1%	29.9%	14.0%	38.6%	33.3%	
One	23.3%	15.8%	20.1%	16.3%	24.7%	22.5%	
Two	15.3%	2.6%	18.2%	11.6%	14.9%	17.4%	
Three	8.7%	10.5%	7.8%	7.0%	8.0%	10.3%	
Four or more	17.3%	50.0%	24.0%	51.2%	13.8%	16.5%	*X*^2^ = 122.675***
SF-12 rating: Fair/poor	26.3%	34.7%	25.9%	38.1%	24.8%	27.7%	*X*^2^ = 10.769

*Note:* The information in this table is based the self-reported survey data. The sample size in this table reflect the number of participants who were able to be coded on each item. Some responses could not be coded because of the amount and quality of the information. The sample sizes ranged from ChildSOs: 30–77; AdultSOs: 52–160; Age-PolySOs: 23–43; Violent: 451–1269; Other: 194–552

^1^ Data from 2001 and 2009 surveys

^*^p < .05, ^**^p < .01, ^***^p < .001

### Criminal careers

*All crime*. There was a significant overall difference between the offender groups in terms of age at first Offence, frequency of offending, and variety (range of crime types committed) of offending (Table 3). Post-hoc comparisons showed that with the exception of ChildSOs (*p* = .119), Age-PolySOs were significantly older than all other offender groups at the time of their first crime (*p*s < .001). Violent offenders were significantly younger at the time of their first offence compared to all other groups (*p*s < .001). Violent offenders also had the highest number of crimes in their offending histories (*M =* 17.46 offences, *SD* = 17.27) of all groups (*p*s < .001), but there were no differences between the ChildSOs, AdultSOs, Age-PolySOs and Other offenders (*p*s > .717). AdultSOs had the highest variety of crimes in their offending histories as they committed all three different offence types (*M =* 2.36, *SD* = 0.80)(*p*s < .001), while ChildSOs, Age-PolySOs, and Violent offenders appeared to equally be more specialised in their offending (*p*s < .765).

**Table 3:** Criminal career parameters in adulthood

	Total Sample (*n* = 2106)	ChildSO (*n* = 76)	AdultSO (*n* = 153)	Age-PolySO (*n* = 43)	Violent (*n* = 1269)	Non-violent (*n* = 552)	*F (df); X*^2^(*df*)
Any Offending							
Age at first charge	26.31 (11.80)	35.04 (15.38)	29.48 (12.61)	42.16 (12.16)	23.11 (11.74)	31.16 (11.74)	*F*(4, 1836) = 81.484***
Frequency	13.91 (15.70)	9.32 (13.23)	9.62 (12.01)	8.77 (6.79)	17.46 (17.27)	7.96 (10.39)	*F*(4, 2096) = 45.259***
Variety	1.75 (.57)	1.84 (.85)	2.36 (.80)	1.70 (.80)	1.88 (.37)	1.27 (.45)	*F*(4, 2096) = 234.302***
Non-violent crimes							
Participation (%)	87.2%	51.3%	75.3%	44.2%	86.6%	100%	*X*^*2*^(4) = 259.045***
Age at first charge	26.37 (11.52)	31.95 (15.81)	29.72 (13.11)	43.21 (13.75)	23.39 (9.68)	31.79 (11.83)	*F*(4, 1713) = 66.368***
Frequency	13.13 (14.23)	12.13 (14.66)	8.89 (10.77)	7.36 (7.14)	16.39 (15.38)	7.98 (10.39)	*F*(4, 1726) = 37.366***
Specialization	78.37 (21.81)	50.60 (23.61)	55.47 (19.98)	48.72 (23.72)	70.36 (18.26)	100 (0)	*F*(4, 1690) = 427.631***
Violent crimes							
Participation (%)^2^	66.8%	32.9%	61.0%	25.6%	100%	–	*X*^*2*^(3) = 810.027***
Age at first charge	25.67 (10.35)	26.79 (11.44)	28.40 (10.5)	39.45 (11.18)	25.29 (10.20)	–	*F*(4, 1249) = 9.426***
Frequency	3.63 (3.48)	1.16 (2.33)	1.64 (2.59)	44 (1.12)	4.13 (3.48)	–	*F*(4, 1538) = 55.443***
Specialization	36.74 (32.21)	6.93 (12.99)	12.05 (15.06)	3.77 (8.56)	42.72 (31.96)	–	*F*(4, 1510) = 95.967***
Sexual crimes							
Participation (%)^3^	13.0%	100%	100%	100%	–	–	*–*
Age at first charge		39.00 (16.04)	33.74 (12.40)	47.84 (11.23)	–	–	*F*(2, 194) = 17.193 ***
Frequency	2.84 (2.89)	2.93 (2.81)	1.94 (1.57)	5.91 (4.27)	–	–	*F*(4, 269) = 41.369***
Specialization	59.43 (38.75)	70.14 (37.80)	48.20 (37.53)	80.21 (30.50)	–	–	*F*(2, 269) = 17.342***

^1^ A total of 13 participants were removed from analyses due to missing information

^2^ Given the method of classification, men who had violent offences (without sexual offence) before or at the time of the survey were classified as violent

^3^ Given the method of classification, men who had a sexual offence before or at the time of the survey were classified as sex offenders

^*^p < .05, ^**^p < .01, ^***^p < .001

**Fig. 2:**
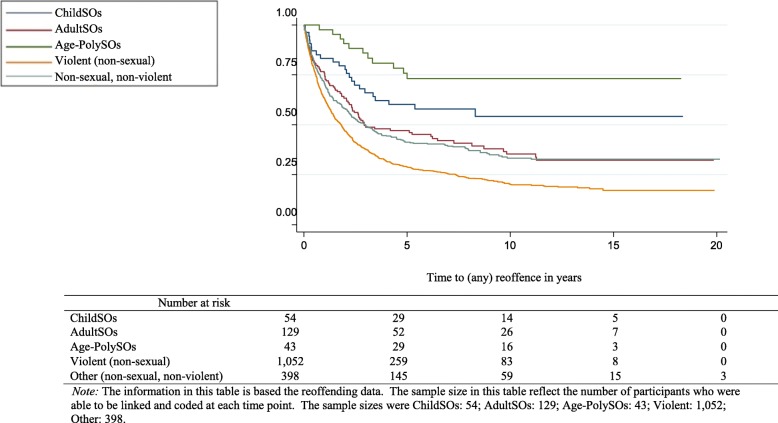
Kaplan-Meier survival curves for the offender groups (at each 5 years)

Crude incidence rates for re-offending were calculated by the offender groups. ChildSOs and Age-PolySOs were the least likely to re-offend with rates of 7.66 per 100 person-years (95% CI: 5.22–11.25) and 3.03 per 100 person-years (95% CI: 1.76–5.21) respectively. The re-offending rates for Other offenders was 8.46 per 100 person-years (95% CI: 7.12–10.00), for AdultSOs it was 11.98 per 100 person-years (95% CI: 9.61–14.94), and for Violent offenders was 23.71 per 100 person-years (95% CI: 22.20–25.31). The median number of years between the release date from custody in which the survey was completed to the date of the next offence or end study date varied between the groups in which analyses could be completed. Violent offenders were found to re-offend quicker than the remaining groups at 1.79 years (95% CI: 1.51–1.98), followed by AdultSOs at 2.92 years (95% CI: 2.31–7.26) and Other offenders at 2.99 years (95% CI: 2.29–3.76). Survival curves illustrate the difference in the time to the next offence from the survey custodial episode and the next offence date (*X*^2^ = 1285.27, *p* < .001) (Fig. 2).

#### Non-sexual, non-violent offending

There were significant overall differences in the age of first non-sexual, non-violent crime, frequency of non-sexual, non-violent crimes, and specialisation in this offending (Table 3). Post-hoc comparisons showed that with the exception of ChildSOs (*M =* 12.12, *SD* = 14.66)(*p* = .725), Violent offenders (*M* = 16.39, *SD* = 15.38) had the highest frequency of non-sexual, non-violent crimes compared to the other groups (*p*s ≤ .004). Non-sexual, non-violent crimes accounted for approximately half of all crimes in the three sex offender subgroups (*p*s > .980), and these offenders showed the least specialisation in these crimes relative to the non-sex offender groups (*p*s ≤ .045). Other offenders showed the highest level of specialisation in other crimes compared to all other groups (*p*s < .001), which is a function of the method of classification.

#### Violent offending

There were significant overall differences in the age of first violent non-sexual crime, frequency of violent crime, and specialisation in violent offending. As might be expected post-hoc comparisons showed that Violent offenders had the highest frequency of violent crimes (*M =* 4.13, *SD* = 3.48) of all groups (*p*s < .001), followed by AdultSOs (*M =* 1.64, *SD* = 2.59) who had significantly more violent crimes than Age-PolySOs (*M =* .44, *SD* = 1.12)(*p* < .001). AdultSOs (12.1%) were significantly more likely to specialise in violent crimes than ChildSOs (6.9%)(*p* = .050) and Age-PolySOs (3.8%)(*p* < .001).

#### Sexual offending

There were significant overall differences in the age of first sexual crime, frequency, and specialisation in sexual offending (Table 3). Post-hoc comparisons showed that Age-PolySOs displayed the highest frequency of sexual crimes (*M =* 5.91, *SD* = 4.27)(*p*s < .001), followed by ChildSOs (*M =* 2.93, *SD* = 2.81) and lastly AdultSOs (*M =* 1.94, *SD* = 1.57)(*p* = .014). In terms of specialisation, AdultSOs had the smallest proportion of sexual crimes in their offending histories compared to the other sex offender subgroups (*p*s < .001).

## Discussion

This study described the data linkage process and classification of sex offenders recruited as part of three waves of a large survey Australian inmate health survey. Data-linkage with an administrative collection of court outcomes enabled access to historical offending records and a more accurate identification of men with histories of sexual offending. Furthermore, the categorisation of sex offenders into subgroups was found to be important as differences were identified in terms of demographic characteristics, health status, and criminal careers.

The surveys classified participants based on the index or most serious offence for the custodial episode when the survey was conducted. The index or most serious offence for the current incarceration episode adopts a static perspective of offending. Classifying participants based on this one point in time may inadequately reflect offending behavior over the criminal career [27]. By using longitudinal historical data rather than only cross-sectional, an additional 35.4% of men with histories of sexual offences were identified. These are important findings and have implications for reporting both criminological and epidemiological research as it demonstrates the value of examining criminal histories before classifying offenders into groups.

The sex offenders identified by looking into their criminal histories (‘historical sex offenders’) were compared to the sex offenders identified at the time of the survey. Historical sex offenders were younger at the time of the survey, had more extensive imprisonment and juvenile offending histories, and different health needs. These findings may be reflective of potentially comparing these two groups (‘historical’ vs. ‘current’) at different points in time during the criminal careers. For example, the criminal careers literature suggests that many sex offenders are convicted (and subsequently serve custodial sentences) for non-sexual crimes [2, 19, 20]. Furthermore, many of the observed differences between the ‘historical sex offenders’ and the ‘current sex offenders’ were not consistent across sex offender subgroups and appear attributable to the differences among the ChildSO groups. This may suggest that AdultSOs tend to have a high frequency in offending, as well as variety in offending over the life course. Previous research has reported similar findings [11], with AdultSOs being identified as resembling the criminal careers of Violent offenders [28].

The current sample of men who committed sexual offences were differentiated into subgroups using the victim-age classification. Differences between the sex offender subgroups were found among the demographic characteristics, summary health variables, and criminal career parameters. These findings were consistent with the existing literature [2, 9, 12, 13] and calls into question the practice of lumping sex offenders together. Lumping appears to limit the opportunity to identify the unique demographic and descriptive characteristics including medical and mental health histories, as well as offending patterns. For example, AdultSOs were found to have demographic characteristics and criminal careers that were more similar to the non-sex offender groups than the ChildSO and Age-PolySO groups. AdultSOs were also found to re-offend at a higher rate and much quicker than the ChildSOs and Age-PolySOs, resembling a re-offending rate similar to the non-sex offender groups. Overall, violent offenders were found to have the highest and quickest rate of re-offending compared to the other groups.

A significant finding from this study is that the Age-PolySOs appeared to represent a distinct offender group. These offenders reported higher academic and professional achievement, were least likely to be single or report having children, and least likely to report juvenile offending histories compared to the other groups. In terms of their criminal careers, Age-PolySOs were the oldest of all the groups at the time of their first recorded offence for other (non-sexual, non-violent) crimes, violent crimes, and sexual crimes. Furthermore, they appeared much more specialised in their offending compared to ChildSOs and AdultSOs as they committed fewer other and violent crimes, but the most sexual crimes. While the finding that they were more specialised in sexual offending is not surprising given that Age-PolySOs by definition have at least two sexual crimes, these results suggests that Age-PolySOs are unique in terms of their demographic characteristics and criminal career parameters.

Differences in the health status among the sex offender subgroups and non-sex offender groups were also identified. While it is well documented that prisoners are beset by poor health [23, 29, 30], comparatively little is known about the health and well-being of sex offenders. We found that ChildSOs and Age-PolySOs had the poorest health of all offender groups, with half reporting four or more chronic physical health conditions. Further research is needed to identify the physical and mental health needs of sex offenders, and bridge the gap between public health and criminal justice. Identifying whether the physical health of sex offenders or health service usage is associated with recidivism will be of benefit to both clinicians and researchers working with this population.

### Limitations

Similar to the majority of studies on sexual reoffending (41% of studies in [7]; 53% in [31]), this study used an official data source (finalised court matters) to determine offending. Official data may minimise the full extent of an offender’s criminal behaviour [27, 32]. For example, a single charge (or conviction) does not necessarily reflect the number of crime events. Furthermore, multiple charges (or convictions) may involve a single victim on a single occasion. The criminal career parameters presented here should be interpreted accordingly. The official data used in this study limited the ability to explore the utility of more detailed sex offender classifications (e.g., victim-gender and victim-offender relationship). Relevant details may be attained by self-report from the offender, other official records (e.g., Police Facts Sheets), and/or psychiatric assessments.

## Conclusions

This study adopted a retrospective cohort design using data linkage to identify a cohort of men with histories of sexual offending recruited as part of an Australian inmate health survey. Data linkage offers the opportunity to more accurately identify and classify sex offenders into subgroups relative to cross-sectional data, which holds implications for epidemiological and survey research. Furthermore, the differentiation of sex offenders into subgroups is important to describe their unique demographic and descriptive characteristics, including medical and mental health histories, as well as criminal career parameters.

## Supplementary information

**Additional file 1.** Supplementary Material.

## Data Availability

The data generated and/or analysed during the current study are not publicly available as this was a condition of release of data to research by source data custodians. The authors take responsibility for the integrity of the data, the accuracy of the data analyses, and have made every effort to avoid inflating statistically significant results.

## Abbreviations

AdultSOs: Refers to ‘adult sex offenders’ or men who offended against adults only
ChildSOs: Refers to ‘child sex offenders’ or men who sexually offended against children only; and
Age-PolySOs: Refers to ‘age-polymorphous sex offenders’ or men who offended against both children and adults

## Footnotes

1 There was a total of 2831 participants in the three health surveys. For this study, the women (*n* = 504) who participated were excluded.

2 After the original linkage, data for a cohort of participants (*n* = 506) was collected from the Re-Offending Database and were available until July 2016.
