# Supplementary material for: Implications of sex offender classification on reporting demographic characteristics, health, and criminal careers: results from an Australian jurisdiction
Source: BMC Med Res Methodol. 2020 Apr 28;20:97. doi: 10.1186/s12874-020-00960-w (PMC7189498; doi:10.1186/s12874-020-00960-w)
Supplement: Supplementary file 1 — Additional file 1. Supplementary Material. [file 12874_2020_960_MOESM1_ESM.docx]

**Supplementary materials**

Below is a description of all post-hoc comparisons written in prose.

**Demographics**

Post-hoc comparisons showed that Age-PolySOs were the older than ChildSOs (*p*=.015) followed by the remaining three groups (*p*s<.001). ChildSOs were older than the AdultSOs, Violent, and Other offenders (*p*s≤.001). AdultSOs were older than Violent offenders (*p*<.001), but were similar in age to Other offenders (*p*=1.000). Violent offenders were significantly younger than Other offenders (*p*<.001).

There was a significant overall difference in the number of prior imprisonments. ChildSOs had the least number of prior imprisonments compared to the other groups (*p*s<.001), with the exception of Age-PolySOs (*p*=1.000). AdultSOs, Age-PolySOs, Violent, and Other offenders all had a similar number of prior imprisonments (*p*s>.126).

**Criminal career parameters in adulthood**

*Any crime.* Post-hoc comparisons showed that with the exception of ChildSOs (*p*=.119), Age-PolySOs were significantly older than all other offender groups at the time of their first offence (*p*s<.001). ChildSOs were significantly older than Violent offenders (*p*<.001) but not AdultSOs and Other offenders (*p*s>.194). AdultSOs and Other offenders were similar in terms of their age (*p*s=.857). Violent offenders were significantly younger at the time of their first offence compared to all other groups (*p*s<.001). Violent offenders had the highest frequency of crimes in their offending histories (*M*=17.46 offences, *SD*=17.27) of all groups (*p*s<.001). There were no significant differences between the ChildSOs, AdultSOs, Age-PolySOs and the Other offenders (*p*>.717). AdultSOs had the highest variety of crimes in their offending histories (*M*=2.39, *SD*=0.79) compared to all other groups (*p*s<.001). ChildSOs, Age-PolySOs, and Violent offenders appeared to equally be more specialised in their offending (*p*s<.765). Other offenders had the least amount of variety in their offending (*p*s<.013).

*Non-sexual, non-violent crime:* ChildSOs were significantly older than Violent offenders at their first recorded Other crime (*p*=.020). ChildSOs were slightly younger than Age-PolySOs but this difference was not statistically significant (*p*=.081). There were no statistically significant differences between ChildSOs and AdultSOs or Other offenders. AdultSOs were older than Violent offenders (*p*<.001). Age-PolySOs were older than AdultSOs (*p*=.006), Violent offenders (*p*<.001), and Other offenders (*p*=.020) at their first recorded Other crime. Other offenders were significantly older than Violent offenders (*p*<.001).

With the exception of ChildSOs (*M*=12.12, *SD*=14.66)(*p*=.725), Violent offenders (*M*=16.39, *SD*=15.38) had the highest frequency of other crimes compared to the other groups (*p*s<.004). There were no statistically significant differences in the number of other crimes in the offender histories between ChildSOs, AdultSOs, PolySOs, and Other offenders (*p*s>.752).

Across all three sex offender subgroups, non-sexual, non-violent crimes accounted for approximately half of all crimes in their offending histories (ChildSOs=50.6%; AadultSOs=55.5%; Age-PolySOs=48.7%) and these groups did not significantly differ in post-hoc comparisons (*p*s>.993). Violent offenders (70.36%) showed a higher level of specialisation in Other crimes in their offending histories (*p*s<.045) relative to the sex offender subgroups. Other offenders showed the highest level of specialisation in other crimes compared to all other groups (*p*s<.001), which is a function of the method of classification.

*Violent (non-sexual) crime*: Age-PolySOs were on average older at the age of first violent (non-sexual) crime than the ChildSOs and Violent offender groups (*p*s<.032) but not the AdultSOs (*p*s<.051). ChildSOs were similar in age to the AdultSOs (*p*=.990), while the violent offenders were significantly younger than the AdultSOs (*p*=.039) but not the ChildSOs (*p*=.989). The violent offender group had the highest frequency of violent crimes (*M*=4.13, *SD*=3.48) compared to any of the sex offender subgroups (*p*s<.001). While ChildSOs (*M*=1.16, *SD*=2.33) were similar to the other sex offender subgroups (*p*s>.145), AdultSOs (*M*=1.64, *SD*=2.59) had significantly more violent crimes than Age-PolySOs (*M*=.44, *SD*=1.12)(*p*<.001). In terms of specialisation in violence, Violent offenders (42.7%) had the highest proportion of violent crimes in their offending histories compared to the sex offender groups (*p*s<.001). AdultSOs (12.1%) were significantly more likely to specialise in violent crimes than ChildSOs (6.9%)(*p*=.050) and Age-PolySOs (3.8%)(*p*<.001). ChildSOs and Age-PolySOs had a similar proportion of violent crimes in their offending histories (*p*=.514).

*Sexual crime:*  Age-PolySOs were the oldest of the sex offender subgroups at first sexual crime (*p*s<.006), followed by ChildSOs who were slightly older than AdultSOs but this difference was not statistically significant (*p*=.116). Age-PolySOs also displayed the highest frequency of sexual offences (*M*=5.91, *SD*=4.27) compared to the other subgroups (*p*<.001), while ChildSOs (*M*=2.93, *SD*=2.81) had significantly more sexual crimes than AdultSOs (*M*=1.94, *SD*=1.57)(*p*=.14). Finally, Age-PolySOs were characterised by the highest proportion of sexual offences in their offending histories (80.2%) followed by ChildSOs (70.1%), however this difference was not statistically significant (*p*=.310). AdultSOs (48.2%) had the least specialisation in sexual offending compared to the other subgroups (*p*s<.001).
